# Incremental genetic K-means algorithm and its application in gene expression data analysis

**DOI:** 10.1186/1471-2105-5-172

**Published:** 2004-10-28

**Authors:** Yi Lu, Shiyong Lu, Farshad Fotouhi, Youping Deng, Susan J Brown

**Affiliations:** 1 Dept. of Computer Science, Wayne State University, Detroit, MI 48202, USA; 2 Department of Biological Sciences, the University of Southern Mississippi, Hattiesburg 39406, USA; 3 Division of Biology, Kansas State University, Manhattan, KS 66506, USA

## Abstract

**Background:**

In recent years, clustering algorithms have been effectively applied in molecular biology for gene expression data analysis. With the help of clustering algorithms such as K-means, hierarchical clustering, SOM, etc, genes are partitioned into groups based on the similarity between their expression profiles. In this way, functionally related genes are identified. As the amount of laboratory data in molecular biology grows exponentially each year due to advanced technologies such as Microarray, new efficient and effective methods for clustering must be developed to process this growing amount of biological data.

**Results:**

In this paper, we propose a new clustering algorithm, *Incremental Genetic K-means Algorithm (IGKA)*. IGKA is an extension to our previously proposed clustering algorithm, the Fast Genetic K-means Algorithm (*FGKA*). IGKA outperforms FGKA when the mutation probability is small. The main idea of IGKA is to calculate the objective value Total Within-Cluster Variation (TWCV) and to cluster centroids incrementally whenever the mutation probability is small. IGKA inherits the salient feature of FGKA of always converging to the global optimum. C program is freely available at

**Conclusions:**

Our experiments indicate that, while the IGKA algorithm has a convergence pattern similar to FGKA, it has a better time performance when the mutation probability decreases to some point. Finally, we used IGKA to cluster a yeast dataset and found that it increased the enrichment of genes of similar function within the cluster.

## Background

In recent years, clustering algorithms have been effectively applied in molecular biology for gene expression data analysis (see [1] for an excellent survey). With the advancement in Microarray technology, it is now possible to observe the expression levels of thousands of genes simultaneously when the cells experience specific conditions or undergo specific processes. Clustering algorithms are used to partition genes into groups based on the similarity between their expression profiles. In this way, functionally related genes are identified. As the amount of laboratory data in molecular biology grows exponentially each year due to advanced technologies such as Microarray, new efficient and effective methods for clustering must be developed to process this growing amount of biological data.

Among the various clustering algorithms, K-means [2] is one of the most popular methods used in gene expression data analysis due to its high computational performance. However, it is well known that K-means might converge to a local optimum, and its result is subject to the initialization process, which randomly generates the initial clustering. In other words, different runs of K-means on the same input data might produce different solutions.

A number of researchers have proposed genetic algorithms [3-6] for clustering. The basic idea is to simulate the evolution process of nature and evolve solutions from one generation to the next. In contrast to K-means, which might converge to a local optimum, these genetic algorithms are insensitive to the initialization process and always converge to the global optimum eventually. However, these algorithms are usually computationally expensive which impedes the wide application of them in practice such as in gene expression data analysis.

Recently, Krishna and Murty proposed a new clustering method called *Genetic K-means Algorithm (GKA) *[7], which hybridizes a genetic algorithm with the K-means algorithm. This hybrid approach combines the robust nature of the genetic algorithm with the high performance of the K-means algorithm. As a result, GKA will always converge to the global optimum faster than other genetic algorithms.

In [8], we proposed a faster version of GKA, FGKA that features several improvements over GKA including an efficient evaluation of the objective value TWCV (Total Within-Cluster Variation), avoiding illegal string elimination overhead, and a simplification of the mutation operator. These improvements result that FGKA runs 20 times faster than GKA [9]. In this paper, we propose an extension to FGKA, *Incremental Genetic K-means Algorithm (IGKA) *that inherits all the advantages of FGKA including the convergence to the global optimum, and outperforms FGKA when the mutation probability is small. The main idea of IGKA is to calculate the objective value TWCV and to cluster centroids incrementally. We then propose a *Hybrid Genetic K-means Algorithm *(HGKA) that combines the benefits of FGKA and IGKA. We show that clustering of microarray data by IGKA method has more tendencies to group the genes with the same functional category into a given cluster.

## Results

Our experiments were conducted on a Dell PowerEdge 400SC PC machine with 2.24G Hz CPU and 512 M RAM. Three algorithms, FGKA, IGKA and HGKA algorithm were implemented in C language. GKA has convergence pattern similar to FGKA and IGKA, but its time performance is worse than FGKA, see [9] for more details. In the following, we compare the time performance of FGKA and IGKA along different mutation probabilities, and then we compare the convergence property of four algorithms, IGKA, FGKA, K-means and SOM (Self Organizing Map). At the end, we check how we can combine IGKA and FGKA algorithm together to obtain a better performance.

### Data sets

The two data sets used to conduct our experiments are serum data, *fig2data*, introduced in [11]and yeast data, *chodata*, introduced in [2]. The *fig2data *data set contains expression data for 517 genes. Each gene has 19 expression data ranges from 15 minutes to 24 hours. In other words, the number of features *D *is 19. According to [11], 517 genes can be divided into 10 groups. The *chodata *is a yeast dataset, composed of expression data for 2907 genes and the expression data for each gene ranges 0 minutes to 160 minutes, which means that the number of features D is 15. According to the description in [2], the genes can be divided into 30 groups. Since the IGKA is a stochastic algorithm, for each experiment in this study, we obtain the results by averaging 10 independent run of the program. The mutation probability, the generation number, the population number all affect the performance and convergence of FGKA and IGKA. The detailed discussion of the parameters setting can be found in [8]. In this paper, we simply adopt the result in [8], the population number is set to 50, and the generation number is set to 100. These parameter setting are safe enough to guarantee the algorithm converge to the optima.

### Comparison of IGKA with FGKA on time performance

As indicated in the implementation section, the mutation probability has great impact on IGKA algorithm. We check the performance impact on IGKA in this section, and the convergence in the next section. Figure 2 shows the time performance results for these two algorithms. We can see that when the mutation probability increases, the running time increases accordingly for both algorithms. However, when the mutation probability is smaller than some threshold (0.005 for *fig2data*, and 0.0005 for *chodata*), IGKA has a better performance. Figure 2 also indicates the thresholds vary from one dataset to another. In order to achieve better performance of IGKA in large data set, mutation probability may need to be set to smaller than that in small data set. For example, in larger data set *chodata*, we should set the mutation probability to 0.0005 to have IGKA outperform FGKA. On the other hand, in order to have IGKA outperform than FGKA, we only need to set the mutation probability to 0.005 in the small data set *fig2data*. In general, the threshold value depends on the number of patterns and the number of features in the data set. It is easy to understand that the performance gained in IGKA is mainly dependent on how many patterns change their cluster memberships. So, in a large data set, even small number of mutation probability may cause many patterns change their cluster memberships.

### Comparison of IGKA with FGKA, K-means and SOM on convergence

Figures 3(A) and 3(B) show the convergence of IGKA versus FGKA across different mutation probabilities based on *fig2data *and *chodata*, respectively. These two algorithms have similar convergence results. When the mutation probability changes in these two data sets, it has little impact on these two algorithms during the range that is given in Figure 3, except for the case when the mutation probability is too large. It gives an opportunity to choose IGKA with better performance without losing the convergence benefit.

We also make an interesting comparison of IGKA with FGKA, K-means and SOM on TWCV convergence. We treat each algorithm as a black box. Two data sets, the *fig2data *and *chodata*, are fed into the algorithms, and the clustering results are exported as a text file. We then use an in-house program to calculate the TWCVs for each result. The experiments on K-means and SOM algorithm are conducted on an open source software [12]. As we can see in Table 2, the IGKA and FGKA have almost similar convergence result, and much better than the convergence of K-means algorithm. The TWCV convergence of SOM is much worse than the others although these four algorithms all use Euclidian distance as their measurement. The reason why we do not include another popular clustering algorithm, hierarchical clustering algorithm is because it is hard to define the boundary among the nested clusters, which means we cannot simply define the number of cluster before running the program.

### Combination of IGKA with FGKA

Figure 4 compares three algorithms, IGKA, FGKA and HGKA, based on the running times for 100 iterations. The mutation probability is set to 0.0001 for all three algorithms. It is clearly that the running time for each iteration of FGKA is much stable than others. On the other hand, the running time for IGKA is much higher than FGKA at the beginning because there are a large number of patterns change their cluster belonging during the K-means operator which cause the IGKA spend a lot of computation time. However, the running time for each iteration of IGKA decrease very sharply at late iterations. The HGKA combines the advantage of two algorithms. The turning point when HGKA uses IGKA instead of FGKA as work horse is highly data dependent. In this particular case, we check the computation time every 15 iterations. The result shows that the performance can be really improved by using HGKA when the mutation probability is small.

## Discussion

The clustering results of *chodata *using our IGKA algorithm were evaluated according to the scheme of gene classification of MIPS Yeast Genome Database [13]. We found that genes of similar function were grouped into the same cluster. Table 3 shows 8 main clusters including 16 functional categories of genes. The results are comparable to the data of [2]. The absolute number of ORFs with functional categories in some cluster may not be always higher than Tavazoie's result, but we found that the percentage of the ORF number within functional category of each cluster in the total ORF number of each cluster is usually higher than Tavazoie's result in most cases. For example, they found that there are 40 genes in the functional category of nuclear organization distributed in their cluster 2, in which there are 186 ORFs, so their percentage is 21.5%. But we found there are 50 genes of the same functional category distributed in our cluster 16, in which there are only 133 ORFs, and our percentage is 37.6% that is significantly higher than 21.5%.

Most interestingly, we found a remarkable enrichment of ORFs for the functional category of organization of mitochondria. They are mainly located in two clusters: cluster 3 and cluster 18. Cluster 3 has 156 ORFs in total, and 111 ORFs belong to the category, resulting in a very high percentage, 71.2%. Cluster 18, has 184 ORFs in total, in which there are 105 ORFs belonging to the category and the percentage is 57.1%. The percentage of ORFs within the same function category is only 18.8% in the previous paper. It looks that our IGKA method is more likely to increase the degree of enrichment of the genes within functional categories, and to make more biological sense. We also found a new function category: lipid and fatty isoprenoid metabolism distributed in cluster 25, which was not listed in Tavazoie's paper.

## Conclusions

In this paper, we propose a new clustering algorithm called *Incremental Genetic K-means Algorithm (IGKA)*. IGKA is an extension of FGKA, which in turn was inspired by the Genetic K-means Algorithm (GKA) proposed by Krishna and Murty. The IGKA inherits the advantages of FGKA, and it outperforms FGKA when the mutation probability is small. Since both FGKA and IGKA might outperform each other, a hybrid approach that combines the benefits of them is very desirable. Our experimental results showed that not only the performance of our algorithm is improved but also the clustering result with gene expression data has some interesting biological discovery.

## Methods

The problem of clustering gene expression data consists of *N *genes and their corresponding *N *patterns. Each pattern is a vector of *D *dimensions recording the expression levels of the genes under each of the *D *monitored conditions or at each of the *D *time points. The goal of IGKA algorithm is to partition the *N *patterns into user-defined *K *groups, such that this partition minimizes the Total Within-Cluster Variation (*TWCV*, also called *square-error *in the literature), which is defined as follows.

Let 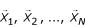 be the *N *patterns, and *X*_*nd *_denotes the *dth *feature of pattern *X*_*n*_(*n *= 1,...*N*). Each partition is represented by a string, a sequence of numbers *a*_1_....*a*_*N*_,, where *a*_*n *_is the number of the cluster that pattern 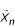 belongs to in this partition. Let *G*_*k *_denote the *kth *cluster and *Z*_*k *_denote the number of patterns in *G*_*k*_. The centroid *c*_*k *_= (*c*_*k*1_, *c*_*k*2_,...,*c*_*kD*_) of cluster *G*_*k *_is defined as 
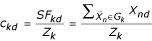
, (*d *= 1,2,...*D*) where *SF*_*kd *_is the sum of the *d*th features of all the patterns in *G*_*k*_. and we use 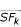 to denote the vector of sum of all patterns in cluster *G*_*k*_.

IGKA maintains a population (set) of *Z *coded solutions, where *Z *is a parameter specified by the user. Each solution, also called a *chromosome*, is coded by a string *a*_1_...*a*_*N *_of length *N*, where each *a*_*n*_, which is called an *allele*, corresponds to a gene expression data pattern and takes a value from {1, 2, ..., K} representing the cluster number to which the corresponding pattern belongs. For example, a_1_a_2_a_3_a_4_a_5_= "33212" encodes a partition of 5 patterns in which, patterns 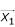 and 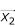 belong to cluster 3, patterns 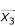 and 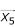 belong to cluster 2, and pattern 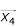 belongs to cluster 1.

### Definition (Legal strings, Illegal strings)

Given a partition *S*_*z *_= *a*_1 _....*a*_*N*_, let *e*(*S*_*z*_) be the number of non-empty clusters in *S*_*z *_divided by *K*, *e*(*S*_*z*_) is called *legality ratio*. We say string *S*_*z *_is *legal *if e(*S*_*z*_) = 1, and *illegal *otherwise.

Hence, an illegal string represents a partition in which some clusters are empty. For example, given *K *= 3, the string a_1_a_2_a_3_a_4_a_5 _= "23232" is illegal because cluster 1 is empty.

**Figure 1:**
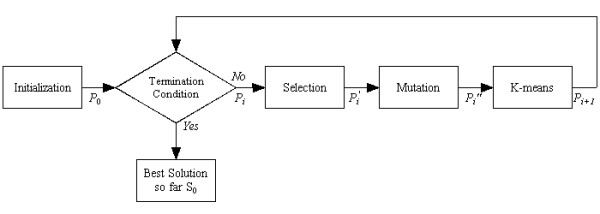
*The flowchart of IGKA algorithm. *It starts with the initialization phase, which generates the initial population *P*_0_. The population in the next generation *P*_*i *+ 1 _is obtained by applying genetic operators on the current population *P*_*i*_. The evolution takes place until a terminating condition is reached. The selection, the mutation and the K-means operator are sequentially used in IGKA.

**Figure 2:**
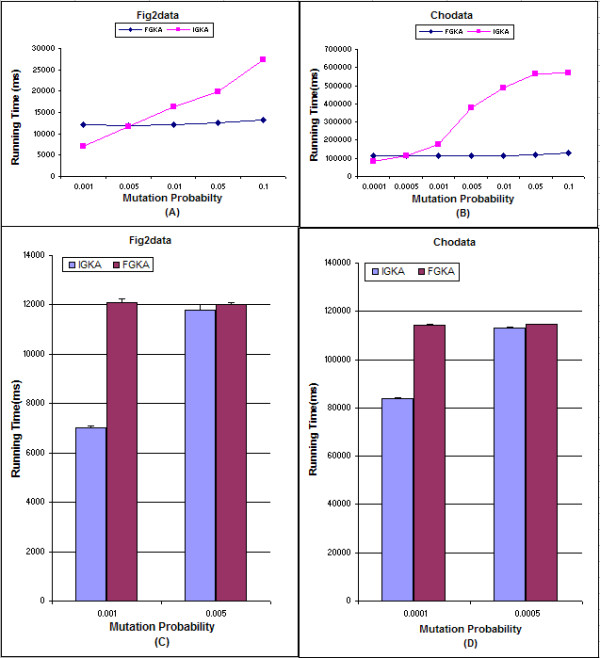
*The impacts of mutation probability on time performance for IGKA and FGKA. *The population size is set to 50; the generation size is set to 100. The mutation probability ranges from 0.001 to 0.1 for *fig2data*, and 0.0001 to 0.1 for *chodata*. (A) shows the running time for FGKA and IGKA on *fig2data*. (B) shows the running time for FGKA and IGKA on *chodata*. (C) shows the average and standard error of running time on *fig2data *when the mutation probability is set to 0.001 and 0.005. (D) shows the average and standard error of running time on *chodata *when the mutation probability is set to 0.0001 and 0.0005. When the mutation probability increases, the running time increases accordingly for both algorithms. However, when the mutation probability is smaller than some threshold (0.005 for *fig2data*, and 0.0005 for *chodata*), the IGKA has better performance. It indicates the thresholds vary from one dataset to another. It mainly depends on the number of patterns and the number of features in the data set.

**Figure 3:**
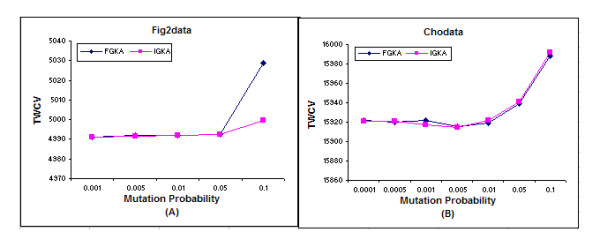
*The impacts of mutation probability on convergence for IGKA and FGKA. *The population size is set to 50; the generation size is set to 100. The mutation probability ranges from 0.001 to 0.1 for *fig2data*, and 0.0001 to 0.1 for *chodata*. (A) shows the convergence with different mutation probability for FGKA and IGKA on *fig2data*. (B) shows the convergence with different mutation probability for FGKA and IGKA on *chodata*. These two algorithms have similar convergence results. When the mutation probability changes in these two data sets, it has little impact on two algorithms during the range that is given in the Figure, except for the case when the mutation probability is too large. It gives an opportunity to choose IGKA with better performance without losing the convergence benefit.

**Figure 4:**
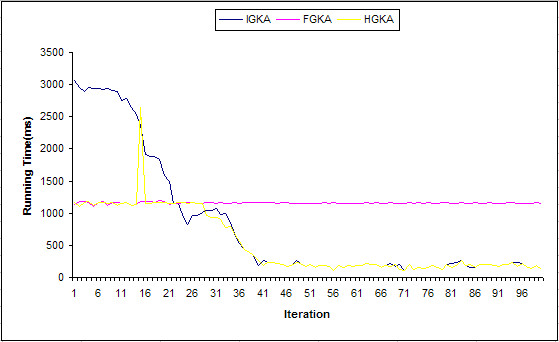
*The performance comparison of IGKA, FGKA and HGKA based on iterations. *The comparison is based on the *chodata *data set, the population number is set to 50 and the mutation probability is set to 0.0001. 100 iterations of three algorithms, IGKA, FGKA and HGKA, are shown in the Figure. The running time for each iteration of FGKA is almost fixed while the running time for IGKA is much higher than FGKA at the beginning and decrease very sharply at late iterations. The HGKA combines the advantage of two algorithms. The turning point in this test case is at iteration 30.

Figure 1 gives the flowchart of IGKA. It starts with the initialization phase, which generates the initial population *P*_0_. The population in the next generation *P*_*i *+ *1 *_is obtained by applying genetic operators on the current population *P*_*i*_. The evolution takes place until a terminating condition is reached. The following genetic operators are used in IGKA: the selection, the mutation and the K-means operator.

### Selection operator

We use the so-called *proportional selection *for the selection operator in which, the population of the next generation is determined by *Z *independent random experiments. Each experiment randomly selects a solution from the current population (S_1_, S_2_, ..., *S*_*z*_) according to the probability distribution (*p*_1_, *p*_2_, ..., *p*_*K*_) defined by 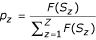
(*z *= 1,...*Z*), where *F*(*S*_*z*_) denotes the fitness value of solution *S*_*z *_with respect to the current population and will be defined in the next paragraph.

Various fitness functions have been defined in the literature [10] in which the fitness value of each solution in the current population reflects its merit to survive in the next generation. In our context, the objective is to minimize the Total Within-Cluster Variation (*TWCV*). Therefore, solutions with smaller *TWCV*s should have higher probabilities for survival and should be assigned with greater fitness values. In addition, illegal strings are less desirable and should have lower probabilities for survival, and thus should be assigned with lower fitness values. We define fitness value of solution *S*_*z*_, *F*(*S*_*z*_) as


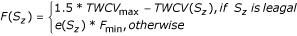


where *TWCV*_*max *_is the maxim *TWCV *that has been encountered till the present generation, *F*_*min *_is the smallest fitness value of the legal strings in the current population if they exist, otherwise *F*_*min *_is defined as 1. The definition of fitness function in GKA [7] paper inspired our definition, but we incorporate the idea of permitting illegal strings by defining the fitness values for them.

The intuition behind this fitness function is that, each solution will have a probability to survive by being assigned with a positive fitness value, but a solution with a smaller TWCV has a greater fitness value and hence has a higher probability to survive. Illegal solutions are allowed to survive too but with lower fitness values than all legal solutions in the current population. Illegal strings that have more empty clusters are assigned with smaller fitness values and hence have lower probabilities for survival. The reason we still allow illegal solution survive with low probability is that we believe the illegal solution may mutate to a good solution and the cost of maintain the illegal solution is very low.

We assume that the *TWCV *for each solution *S*_*z *_(denoted by *S*_*z*_.*TWCV*) and the maximum TWCV (denoted by *TWCV*_*max*_), have already been calculated before the selection operator is applied.

### Mutation operator

Given a solution (chromosome) that is encoded by *a*_1 _....*a*_*N*_, the mutation operator mutates each allele *a*_*n*_(*n *= 1, ..., *N*) to a new value *a*_*n *_(*a*_*n *_might be equal to *a*_*n*_) with probability *MP *respectively and independently, where 0 <*MP *< 1 is a parameter called the *mutation probability *that is specified by the user. The mutation operator is very important to help reach better solutions. From the perspective of the evolutional theory, offsprings produced by mutations might be superior to their parents. More importantly, the mutation operator performs the function of shaking the algorithm out of a local optimum, and moving it towards the global optimum.

Recall that in solution *a*_1 _....*a*_*N*_, each allele *a*_*n *_corresponds to a pattern 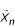 and its value indicates the number of the cluster to which 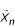 belongs. During mutation, we replace allele *a*_*n *_by *a*_*n*_' for *n *= 1,...,*N *simultaneously, where *a*_*n *_is a number randomly selected from (1,....,K) with the probability distribution (*p*_1_, *p*_2_, ..., *p*_*K*_) defined by:


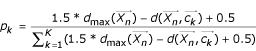


where 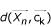 is the Euclidean distance between pattern 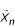 and the centroid *c*_*k *_of the *k*th cluster, and 
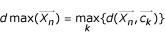
. If the *k*th cluster is empty, then 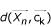 is defined as 0. The bias 0.5 is introduced to avoid divide-by-zero error in the case that all patterns are equal and are assigned to the same cluster in the given solution. Our definition of the mutation operator is similar to the one defined in the GKA paper [7]. However, we account for illegal strings, which are not allowed in the GKA algorithm.

The above mutation operator is defined such that (1) 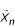 might be reassigned randomly to each cluster with a positive probability; (2) the probability of changing allele value *a*_*n *_to a cluster number *k *is greater if 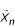 is closer to the centroid of the *k*th cluster *G*_*k*_; and (3) empty clusters are viewed as the closest clusters to 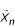. The first property ensures that an arbitrary solution, including the global optimum, might be generated by the mutation from the current solution with a positive probability; the second property encourages that each 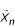 is moving towards a closer cluster with a higher probability; the third property promotes the probability of converting an illegal solution to a legal one. These properties are essential to guarantee that IGKA will eventually converge to the global optimum fast.

### K-means operator

In order to speed up the convergence process, one step of the classical K-means algorithm, which we call *K-means operator (KMO) *is introduced. Given a solution that is encoded by *a*_1 _....*a*_*N*_, we replace *a*_*n *_by *a*_*n*_' for *n *= 1,...,*N *simultaneously, where *a*_*n*_' is the number of the cluster whose centroid is closest to 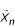 in Euclidean distance. More formally, 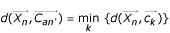


To accommodate illegal strings, we define 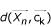 = +∞ if the *k*th cluster is empty. This definition is different from mutation operator, in which we defined 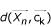 = 0 if the *k*th cluster is empty. The motivation for this new definition here is that we want to avoid reassigning *all *patterns to empty clusters. Therefore, illegal string will remain illegal after the application of KMO.

In the following, we first present FGKA algorithm that is proposed in [9]. We then describe the motivation for IGKA based on the idea of incremental calculation of TWCV and centroids. Finally, we present a hybrid approach that combines the benefits of FGKA and IGKA.

### Fast Genetic K-Means Algorithm (FGKA)

FGKA shares the same flowchart of IGKA given in Figure 1. It starts with the initialization of population *P*_0 _with *Z *solutions. For each generation *P*_*i*_, we apply the three operators, selection, mutation and K-means operator sequentially which generate population 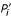, 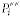, and *P*_*i *+ 1 _respectively. This process is repeated for *G *iterations, each of which corresponds to one generation of solutions. The best solution so far is observed and recorded in *S*_*o *_before the selection operator. *S*_*o *_is returned as the output solution when FGKA terminates.

### Incremental Genetic K-Means Algorithm (IGKA)

Although FGKA outperforms GKA significantly, it suffers from a potential disadvantage. If the mutation probability is small, then the number of allele changes will be small, and the cost of calculating centroids and TWCV from scratch can be much more expensive than calculating them in an incremental fashion. As a simple example, if a pattern 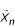 is reassigned from cluster *k *to cluster *k'*, then only the centroids and *WCV*s of these two clusters need to be recalculated. Furthermore, the centroids of these two clusters can be calculated incrementally since the memberships of other patterns have not changed; The *TWCV *can be calculated incrementally as well since the *WCV*s of other clusters have not changed. In the following, we describe how we can calculate *TWCV *and cluster centroids 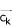 incrementally.

In order to obtain the new centroid 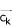, we maintain the difference values of *Z*_*k*_^Δ^, 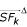 for old solution and new solution when allele changes. With these two values, incremental update of *Z*_*k *_and 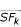 can be achieved as *Z*_*k *_= *Z*_*k *_+ *Z*_*k*_^Δ^, and 
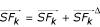
. Then the new centroids for new solution 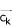 can be achieved by 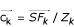.

Similarly, in order to obtain the new *TWCV*, we can maintain a difference value *TWCV*^Δ ^that denotes the difference between old *TWCV *and new TWCV for one solution. It is obvious that *TWCV*^Δ ^is attributed from the difference of new *WCV*_*k *_and old *WCV*_*k *_for cluster k. However, *WCV*_*k *_has to be calculated from scratch since 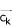 is changed. In this way, TWCV can be updated incrementally as well. Since the calculation of *TWCV *dominates all iterations, our incremental update of *TWCV *will have a better performance when mutation probability is small (which implies a small number of alleles changes). However, if the mutation probability is large, too many alleles change their cluster membership, the maintenance of Z_*k *_^Δ ^and 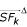 becomes expensive and IGKA becomes inferior to FGKA in performance, as confirmed in the experimental study.

### Hybrid Genetic K-Means Algorithm (HGKA)

The above discussion presents a dilemma – both FGKA and IGKA are likely to outperform each other: when the mutation probability is smaller than some threshold, IGKA outperforms FGKA; otherwise, FGKA outperforms IGKA.

The key idea of HGKA is to combine the benefits of FGKA and IGKA. However, it is very difficult to derive the threshold value, which is dataset dependant. In addition, the running times of all iterations will vary as solutions converge to the optimum. We propose the following solution: we periodically run one iteration of FGKA followed by one iteration of IGKA while monitoring their running times, and then run the winning algorithm for the following iterations until we reach another competition point.

It has been proved in [8] that FGKA will eventually converge to the global optimum. By using the same flowchart and operators, IGKA and HGKA will also converge to the global optimum. We summarize the comparison of various clustering algorithms in Table 1.

## Availability and requirements

IGKA algorithm is available at . The source code and database scheme are freely distributed to academic users upon request to the authors.

## List of abbreviations

WCV: Within-Cluster Variation; TWCV: Total Within-Cluster Variation; IGKA: Incremental Genetic K-means Algorithm; FGKA: Fast Genetic K-means Algorithm; HGKA: Hybrid Genetic K-means Algorithm; ORF: Open Reading Frame.

## Authors' contributions

YL carried out the study and drafted the manuscript. SL and FF designed the algorithms. YD designed the whole project, participated in analyzing gene functional data and wrote part of manuscript. SJB corrected English and helped to interpret the data analysis results.

## Figures and Tables

**Table 1:** Comparison of different algorithms on performance, convergence and stability. Five apporaches are compared based on time performance, convergence and stability. The K-means algorithm has better time performance than any other genetic algorithms, but it suffers from converging to local optimum and initialization dependent. Among the four genetic clustering approaches, Hybrid approach always has better time performance while FGKA performs well when the mutation probability is big, and IGKA performs well when the mutation probability is small. IGKA and FGKA outperform GKA. The convergence of four genetic algorithms has similar results, and all four are independent from the initialization.

	K-means	GKA	FGKA	IGKA	Hybrid
Time	Fastest	Slow	Good when the mutation	Good when the mutation	Good
Performance			probability is large	probability is small	
Convergence	Worse	Good	Good	Good	Good
Stability	Unstable	Stable	Stable	Stable	Stable

**Table 2:** Comparison of different algorithms on TWCV convergence with two data sets. Four algorithms, IGKA, FGKA, K-means and SOM are experimented on the two data set, the *fig2data*, and *chodata*. The TWCVs of IGKA and FGKA algorithm are obtained by averaging 10 individual runs while the generation number is set to 100, the population number is set to 50, the mutation probability is set to 0.005 for *fig2data*, and 0.0005 for *chodata*. The TWCV of K-means algorithm is obtained by averaging 20 individual runs. The TWCV of SOM is obtainedby 8 individual runs with different setting on X and Y dimension. The IGKA and FGKA algorithms have better TWCV convergence than the K-means and SOM.

Algorithms	Fig2data	Chodata
IGKA (Average of 10 individual runs with generation 100, population 50, mutation probability 0.005 in *fig2data*, and 0.0005 in *chodata*)	4991.53889	16995.7
FGKA (Average of 10 individual runs with generation 100, population 50, mutation probability 0.005 in *fig2data*, and 0.0005 in *chodata*)	4992.13889	16995.4
K-means (Average of 20 individual runs)	5154.21434	17374.6758
SOM (Average of 8 individual runs with different setting)	24805.3661	21660.9049

**Table 3:** Distribution of ORF function categories in the clusters. Chodata set was clustered using IGKA algorithm. We identified the gene distribution of different functional categories into different clusters. The function categories were divided according to MIPS (Mewes et al., 2000). The total number of ORFs in each function category was indicated in parentheses. The cluster number to which the genes were grouped is denoted as "Cluster" column. The ORF number in each cluster is denoted as "Total". The ORF number within each functional category is denoted as "Function ORFs". The percentage of the ORF number within functional category of each cluster in the total ORF number of each cluster is denoted as "Percentage (%)".

Cluster	MIPS functional category	Total	Function ORFs	Percentage(%)
1	Mitotic cell cycle and cycle control(352)	86	24	27.9
	Budding, cell polarity, filament form(170)		8	9.3
3	Organization of mitochondrion(366)	156	111	71.2
	Respiration(88)		10	6.4
	Nitrogen and sulpur metabolism(67)		9	5.6
16	Organization of nucleus(774)	133	50	37.6
17	Ribosome biogenesis(215)	88	50	56.8
	Organization of cytoplasm(554)		31	35.2
18	Organization of mitochondrion(366)	184	105	57.1
25	DNA synthesis and replication(94)	164	23	14
	DNA recombination and DNA repair(153)		11	6.7
	Lipid and fatty isoprenoid metabolism(213)		9	5.5
29	Organization of nucleus chromosome(44)	93	14	15
	Amino acid metabolism(204)		12	12.9
30	TCA pathway or Krebs cycle(25)	92	7	7.6
	C-compound, carbohydrate metabolism(415)		14	15.2
